# Evaluation of Altered Functional Connections in Male Children With Autism Spectrum Disorders on Multiple-Site Data Optimized With Machine Learning

**DOI:** 10.3389/fpsyt.2019.00620

**Published:** 2019-09-20

**Authors:** Giovanna Spera, Alessandra Retico, Paolo Bosco, Elisa Ferrari, Letizia Palumbo, Piernicola Oliva, Filippo Muratori, Sara Calderoni

**Affiliations:** ^1^National Institute for Nuclear Physics (INFN), Pisa Division, Pisa, Italy; ^2^IRCCS Stella Maris Foundation, Pisa, Italy; ^3^Scuola Normale Superiore, Faculty of Sciences, Pisa, Italy; ^4^Department of Chemistry, and Pharmacy, University of Sassari, Sassari, Italy; ^5^National Institute for Nuclear Physics (INFN), Cagliari Division, Cagliari, Italy; ^6^Department of Clinical and Experimental Medicine, University of Pisa, Pisa, Italy

**Keywords:** autism spectrum disorders, children, resting-state fMRI, functional connectivity, machine learning, ABIDE

## Abstract

No univocal and reliable brain-based biomarkers have been detected to date in Autism Spectrum Disorders (ASD). Neuroimaging studies have consistently revealed alterations in brain structure and function of individuals with ASD; however, it remains difficult to ascertain the extent and localization of affected brain networks. In this context, the application of Machine Learning (ML) classification methods to neuroimaging data has the potential to contribute to a better distinction between subjects with ASD and typical development controls (TD). This study is focused on the analysis of resting-state fMRI data of individuals with ASD and matched TD, available within the ABIDE collection. To reduce the multiple sources of heterogeneity that impact on understanding the neural underpinnings of autistic condition, we selected a subgroup of 190 subjects (102 with ASD and 88 TD) according to the following criteria: male children (age range: 6.5–13 years); rs-fMRI data acquired with open eyes; data from the University sites that provided the largest number of scans (KKI, NYU, UCLA, UM). Connectivity values were evaluated as the linear correlation between pairs of time series of brain areas; then, a Linear kernel Support Vector Machine (L-SVM) classification, with an inter-site cross-validation scheme, was carried out. A permutation test was conducted to identify over-connectivity and under-connectivity alterations in the ASD group. The mean L-SVM classification performance, in terms of the area under the ROC curve (AUC), was 0.75 ± 0.05. The highest performance was obtained using data from KKI, NYU and UCLA sites in training and data from UM as testing set (AUC = 0.83). Specifically, stronger functional connectivity (FC) in ASD with respect to TD involve (p < 0.001) the angular gyrus with the precuneus in the right (R) hemisphere, and the R frontal operculum cortex with the pars opercularis of the left (L) inferior frontal gyrus. Weaker connections in ASD group with respect to TD are the intra-hemispheric R temporal fusiform cortex with the R hippocampus, and the L supramarginal gyrus with L planum polare. The results indicate that both under- and over-FC occurred in a selected cohort of ASD children relative to TD controls, and that these functional alterations are spread in different brain networks.

## Introduction

According to the *Diagnostic and Statistical Manual of Mental Disorders*, fifth edition (DSM-5) (1) autism spectrum disorders (ASD) are a heterogeneous set of neurodevelopmental disorders characterized by deficits in social communication and social interaction and the presence of restricted, repetitive behaviors. Updated data on the prevalence of ASD in the United States (Centers for Disease Control and Prevention—CDC) (2) identified 1 in 59 children (1 in 37 boys and 1 in 151 girls) as having ASD. The exact etiopathogenesis of idiopathic ASD is not yet fully established: however, recent evidences point to an interaction between genetic liability and environmental factors in producing early alteration of brain development (3). In this framework, some recent studies have used pattern classification techniques to analyze structural and functional neuroimaging data, in order to highlight brain signatures able to distinguish ASD subjects from controls (4).

Among neuroimaging techniques, resting-state functional magnetic resonance imaging (rs-fMRI) allows to collect brain functional connectivity (FC) data from individuals not engaged in any specific task (5), and thus it is particularly suited to extract information on the functional brain organization of young or non-cooperative or low-functioning ASD subjects (6). In particular, recent rs-fMRI investigations have provided crucial evidence on the disruption of functional networks in individuals with ASD (7–9). However, rs-fMRI findings of subjects with ASD suggested conflicting patterns of FC, with the presence of over FC, under FC and a combination of both (10). Most studies focused on adolescents and adults, where under FC in subjects with ASD has been predominantly observed, and usually found to be related to social impairment (11,). The under-FC pattern involves several brain areas, including the salience network, the default mode network (DMN), and language-related regions (11, 13, 14). Conversely, studies carried out on young children have demonstrated that there is an over-FC pattern, detected at whole-brain level and in subsystems (15), in particular in the default mode, salience, frontotemporal, motor and visual networks (16).

The inconsistent results obtained on adults, adolescents and children suggest that the alteration of FC could be partly ascribed to age. Since ASD has an early developmental origin, it is necessary to focus on childhood to be sure that no age-related compensatory mechanisms have already happened (15). Due to the possible age dependence of FC alterations in ASD, it is important to select a specific age range for the cohort of subjects involved in research studies (17). Furthermore, it has been observed that sex impact on both structural (18–20) and functional (21) brain organization in subjects with ASD. Another factor to consider is eye status during scan, which may introduce FC alterations, in particular at local level (22).

Several investigations analyzed the FC with machine learning methods (17–23). These tools are able to learn relevant differences between a group of subjects affected by a specific condition and a control group of subjects with typical development from a dataset (training set) and make predictions on unknown observations (testing set). As a general rule, the greater the number of subjects used in the training phase, the higher the reliability and generalization ability of the classifier. Large data samples are difficult to acquire in a single site, thus they are often obtained by collecting data from multiple sites. In this case, a classifier is trained on a more representative cohort of subjects, therefore, in principle, it can make more general predictions. However, additional sources of variability may affect multicenter analysis, e.g. slightly different acquisition protocols or participant instructions during image acquisition (23), and it has been observed that classification accuracy for multi-site analysis is lower than single-site results (24). Moreover, the site-dependent information encoded in multi-site data may lead a classifier to learn to distinguish categories of subjects according to confounding parameters instead of relying on differences between subjects related to the diagnostic classes.

We explored in this study the FC of subjects with ASD, exploiting the potential of machine-learning approaches to highlight subtle differences between the FC profile of subjects with ASD and controls.

## Materials and Methods

### Sample Composition

**Figure 1:**
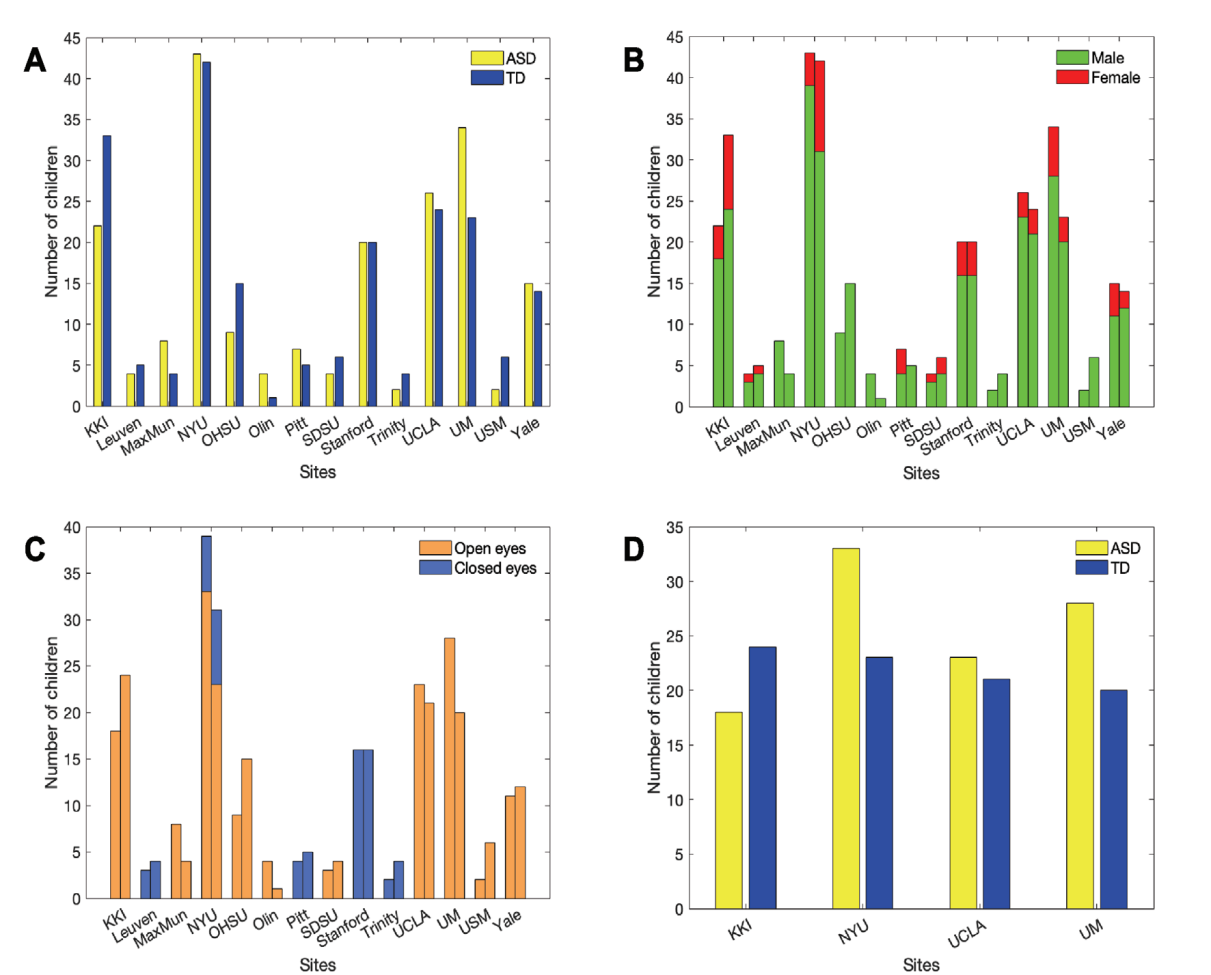
Number of children per ASD and TD groups (yellow = ASD, blue = TD) before **(A)** and after adopting selection criteria regarding sex **(B)**, eye status during scan **(C)** and sample size at each site **(D)**.

We selected a sample of subjects with ASD and controls for our analysis within the publicly-available data sample collected within the Autism Brain Imaging Data Exchange (ABIDE) initiative^1^ (25). The main selection of subjects was carried out on participants’ age: specifically, we focused our analysis on children in the age range of 6.5 to 13 years to reduce the impact of developmental changes during puberty. Several sites contributing to the ABIDE I collection recruited participants below 13 years of age, except Caltech, CMU and SBL (**Figure 1A**).

In addition to age, participants were chosen according to sex, eye status during the scan, and the available number of subjects with the selected characteristics at each site. Only male children were selected since the male sample size is larger than female across sites (**Figure 1B**), in line with the epidemiology of ASD (26). Scans with open eyes were chosen because they are more numerous and with a low risk of sleep during acquisition time, that can represent an additional source of variability that is difficult either to monitor or prevent (22) (**Figure 1C**).

After the previous selections, only the four most populated remaining sites were analyzed (n = 190; ASD = 102 and TD = 88) (**Figure 1D**): Kennedy Krieger Institute (KKI), NYU Langone Medical Center (NYU), University of California, Los Angeles (UCLA), University of Michigan (UM). Furthermore, the groups of subjects from KKI (ASD = 18, TD = 24), NYU (ASD = 33, TD = 23), UCLA (ASD = 23, TD = 21), UM (ASD = 28, TD = 20) were age-matched.

More details about the impact of selection criteria on the classification performance are reported in Supplementary Materials.

**Figure 2:**
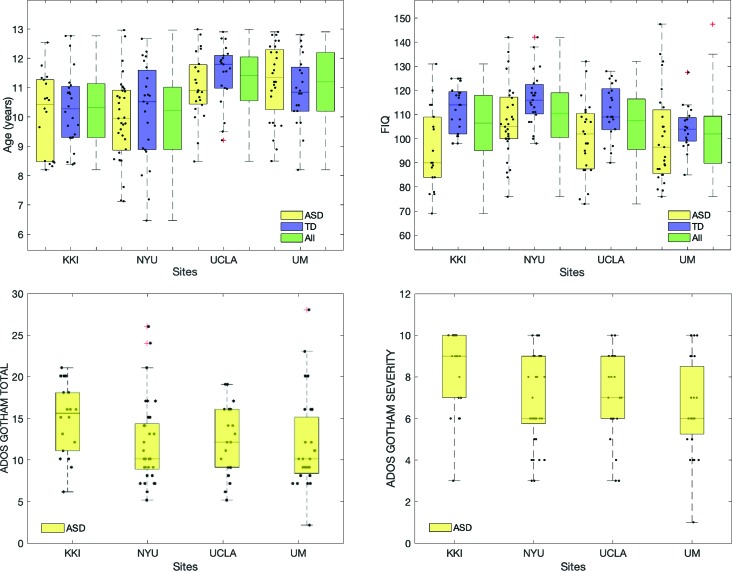
Distributions of age, FIQ, ADOS Gotham total and ADOS Gotham severity scores across sites. Top row: age and FIQ distributions are reported for ASD, TD and all subjects together; bottom row: ADOS total (left) and ADOS Gotham severity (right) scores are shown for children with ASD. Points representing each single subject were overlaid to the box plot. A small random noise has been added on x axis label for each subject in order to make all points visible.

The mean and standard deviation values of age, full scale intelligence quotient (FIQ), ADOS Gotham total and ADOS Gotham severity scores (27) are reported for each site for ASD and TD groups of subjects in **Table 1**. The distributions of clinical and demographic variables are reported in **Figure 2**. The selected site parameters, in terms of vendor, scan duration (28) and diagnostic category are reported in **Table 2**.

**Table 1:** Dataset composition and sample characteristics in KKI, NYU, UCLA and UM sites.

Sites	Subject group, mean ± std [range]		Statistical test	
	ASD	TD	Statistic	p-value
	**Age (Years)**		**t-Test (t)**	
KKI	10.1 ± 1.4 [8.2–12.5]	10.3 ± 1.3 [8.4–12.8]	−0.51	0.62
NYU	10 ± 1.4 [7.1–13]	10.2 ± 1.7 [6.5–12.7]	−0.52	0.61
UCLA	11 ± 1.1 [8.5–13]	11.5 ± 1 [9.2–12.9]	−1.37	0.18
UM	11.2 ± 1.3 [8.5–12.9]	10.9 ± 1.2 [8.2–12.8]	0.92	0.36
	**FIQ**		**Mann-Whitney Test (z)**	
KKI	95 ± 17 [69–131]	112 ± 10 [98–125]	−3.17	<0.001*
NYU	108 ± 16 [76–142]	117 ± 11 [98–142]	−2.58	0.01*
UCLA	100 ± 16 [73–132]	111 ± 11 [90–128]	−2.4	0.02*
UM	101 ± 20^+^ [73–132]	105 ± 9 [85–127]	−1.49	0.14
	**ADOS Gotham total**			
KKI	15 ± 4 [6–21]			
NYU	12± 5 [5–26]			
UCLA	12 ± 4^+^ [5–19]			
UM	12 ± 6^+^ [2–28]			
	**ADOS Gotham severity**			
KKI	8 ± 2 [3–10]			
NYU	7 ± 2 [3–10]			
UCLA	7 ± 2^+^ [3–10]			
UM	7 ± 2^+^ [1–10]			

ASD, autism spectrum disorder; TD, typical developmental control; std, standard deviation; FIQ, full scale intelligence quotient.

t, two group independent t test statistic between ASD and TD groups mean values.

z, two group independent Mann-Whitney test statistic between ASD and TD groups median values.

*Significant differences between mean (or median) ASD and TD groups.

^+^Missing values from some UCLA and UM sites ASD children were removed in calculating the mean and the standard deviation of parameters.

**Table 2:** KKI, NYU, UCLA and UM characteristics in terms of vendor, scan duration and the diagnostic categories.

Sites	Scanner	Time scan (min)	Participants	
			TD	ASD
KKI	Philips	6.33	24	18
NYU	Siemens Allegra	5.9	23	33
UCLA	Siemens Trio Tim	5.8	21	23
UM	GE	9.8	20	28

ASD, autism spectrum disorder; TD, typical developmental control.

### Resting-State fMRI Data

We analyzed the preprocessed data available on the ABIDE preprocessed homepage (29, 30), using the Configurable Pipeline for the Analysis of Connectomes (CPAC) (31), that includes slice-timing, motion correction, intensity normalization, nuisance signal removal (e.g. tissue signals, low-frequency drifts), and registrations. Band-pass filtering and global signal regression strategies were chosen as processing strategies to reduce the impact of physiological noise and global signal, that includes non-neuronal components and fluctuations in neuronal activity (32). Both anatomical and functional atlases were chosen to derive the FC measures; in particular, we chose Harvard-Oxford (HO) and Automatic Anatomical Labeling (AAL) as anatomical templates and Craddock-200 (CC) as functional templates to extract time series from brain regions (33). The timeseries and the information about labels of regions for each atlas are reported on the ABIDE preprocessed homepage in the pipeline section. Further analysis was conducted using the functional Power template obtained by brain-wide graph analysis (34). For this analysis, we extracted the time series from the preprocessed functional images since they are not directly available on the ABIDE preprocessed homepage.

### Functional Connectivity Analysis

For each atlas, the Pearson correlation was calculated between the time series of pairs of regions to obtain a NxN correlation matrix for each subject, where N indicates the number of regions of the selected atlas.

The correlation values were normalized according to Fisher transformation (35), where the number of timepoints is taken into account:

$$Z=\frac{1}{2}\sqrt{n-3}\ln\left(\frac{1+r}{1-r}\right)$$

where n is the number of timepoints of time series and r indicates the Pearson correlation values. From each symmetric FC matrix obtained we used $\frac{N(N-1)}{2}$ non-redundant values as features for the machine learning classification.

### Machine Learning Based Classification

Supervised binary classification of ASD and TD classes was carried out with Support Vector Machines (SVM) (36), which are able to handle noisy and correlated features and can provide better results with respect to other classifiers on dataset with small samples and large number of features (37).

SVM classifiers are able to separate distributions of data in two classes (i.e. ASD and TD) through a hypersurface, described a function according to selected kernel (38). This separation surface is learned from the training set and allows to make predictions on testing set, composed by unknown data. A linear-kernel support-vector-machine (L-SVM) was chosen as it has been demonstrated to provide a more robust performance with respect non-linear kernel SVM when the number of features is large with respect to the number of training cases (39). In addition, L-SVM provide a direct way to interpret the findings: the separating hyperplane is a linear function defined by the weight vectors and an offset. The weights associated with each feature express the direction along which the normalized pairwise correlations differ between two classes: higher weights correspond to more discriminating features; the weight signs allow to identify whether a connection is stronger or weaker in ASD than TD subjects.

Machine-learning based classifiers were implemented to optimize the evaluation of possible altered functional connections in ASD. In particular, the L-SVM classification was carried out on the FC features derived for each parcellation scheme to choose the optimal one among the AAL, HO, CC and Power atlases. In order to reduce the effect of site-specific sources of variability (23), a leave-site out cross-validation scheme was performed: each training set was composed by all the sites except one, that was left out for validation. The classification performance was evaluated in terms of the area under the ROC curve (AUC) (40).

### Significant Connections

In order to identify the most significant connections able to discriminate between ASD and TD children, a permutation test was carried out on the entire dataset of children. This non-parametric technique allows to assign statistical significance to the classification.

A L-SVM classifier was trained on the data after 10,000 permutations of the class labels. The absolute values of the obtained weights were compared with the ones of the classifier trained on the correct labels (38, 39). Through this procedure probability maps were generated and thresholded at three different p-values (p < 0.01, p < 0.005, p < 0.001) in order to identify the most discriminating functional connections and to visualize them at different significance levels.

Depending on the weight signs, the alteration in FC are recognized as over FC, in correspondence to positive model weights, and under FC, in correspondence to negative model weights.

Functional alterations in terms over FC and under FC were analysed in the Mesulam subsystems (25, 41), including the connections both between and within heteromodal, unimodal, paralimbic, limbic, primary, and subcortical regions.

### Statistical Methods and Analysis Tools

Statistical tests were conducted on age and FIQ values to evaluate the matching between the cohorts of ASD and TD children in each site. Specifically, t-test was conducted on age values and Mann-Whitney U-test on not normal FIQ values. The normality of the distributions of age and FIQ values was evaluated by Shapiro-Wilk test.

Furthermore, statistical differences across sites were evaluated through one-way ANOVA, which was applied on the normally-distributed age values, and Kruskal-Wallis test, which was applied on nonnormally-distributed FIQ and ADOS scores, the latter standardized according to Gotham algorithms (27). In particular, we analyzed the ADOS Gotham total score, which is related to social affect and restricted repetitive behaviour, and the ADOS Gotham severity score, which captures the calibrated autism symptom severity. The statistical tests results were corrected using Bonferroni method for multiple comparison correction.

In order to evaluate the significant functional connections different between each site and the other sites combined together, a Mann-Whitney U-test was carried out. In particular the analysis was conducted only on the control children to avoid to include confounding effects related to the disorder. The p-values obtained were corrected using Benjamini-Hochberg false discovery rate (FDR), taking account for the number of false discovery (q ≤ 0.05) (42).

Possible correlations between functional connections showing the most significant group differences and autism symptom severity and overall level of functioning have been investigated according to Spearman rank correlation coefficient. Specifically, the relationships between FIQ and FC values were evaluated in ASD and TD groups separately.

Functional connectivity analysis, classifications, permutation test and cerebral maps representation, and the study of correlations between altered FC values and clinical scores were carried out with Matlab 2017a (The MathWorks, Inc.). In particular, in-house built scripts and functions were developed, and, for the SVM classifier training, the *fitcsvm* matlab function has been used, with the default choice of the *c* parameter – the parameter that regulates the trade-off between having zero training errors and allowing for misclassifications – for the linear-kernel SVM, to avoid running optimization of hyperparameters, which would have required and additional nested cross validation.

### Effect of Site and Other Confounding Parameters

The impact of the site and of the other confounding parameters (e.g. sex, eye status) on the performance in the ASD vs. TD machine-learning based classification was evaluated and reported in the Supplementary Materials. A statistical comparison among the FC maps of TD children obtained at the four different sites was also carried out to highlight the impact of the acquisition site on FC information (see **Supplementary Materials**).

## Results

### Sample Analysis

T-test analysis on age and Mann-Whitney analysis on FIQ values in each site showed that ASD and TD groups are only age-matched whereas no dataset is matched on FIQ, except for the UM sample (**Table 1**). The results of one-way ANOVA and Kruskal-Wallis analyses carried out for each participant’s parameter showed that there are significant differences between two or more sites according to age and FIQ. Multiple comparisons, using Bonferroni correction, were conducted for each parameter to identify which sites were different according those parameters. Both KKI and NYU samples showed statistically significant differences from UCLA and UM samples according to age, whereas only the NYU sample was different from the UM sample according to FIQ (**Table 3**).

**Table 3:** One-way ANOVA/Kruskal-Wallis analysis for each participant’s parameter: age, FIQ, ADOS Gotham total, ADOS Gotham severity. The Bonferroni correction for multiple comparisons has been used. The tests on age and FIQ values have been conducted on the cohorts of subjects including both ASD and TD children of each site.

Variable	N	Statistical test		Group
		Statistic	p value	
Age (years)	48	F = 10.23	<0.001*	KKI-UCLA, KKI-UM, NYU-UCLA, NYU-UM
		
		
FIQ	47	χ^2^ = 9.51	0.02	NYU-UM
ADOS Gotham total	99	χ^2^ = 7.15	0.07	
ADOS Gotham severity	99	χ^2^ = 8.05	0.05	

### Functional Connectivity Measures

**Figure 3:**
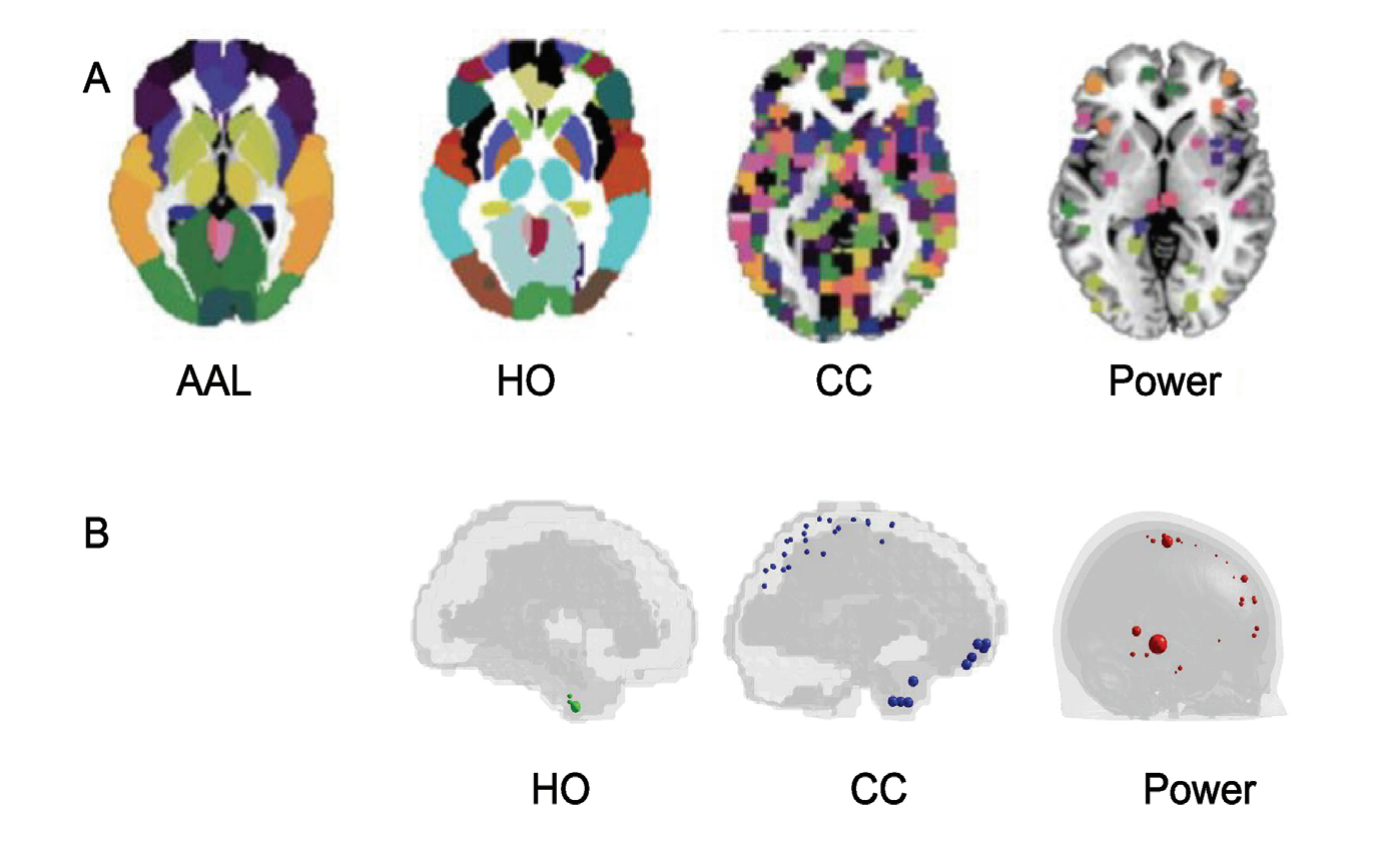
Parcellation schemes used in this analysis **(A)**: Automated Anatomical Labeling (AAL), Harvard-Oxford (HO), Craddock (CC) and Power atlases used in functional connectivity analysis. Regions with null time series obtained in implementing the HO, CC, and Power atlases on data **(B)**. Critical regions are represented as spheres positioned in the centroid of each atlas region, with a radius proportional to the number of subjects presenting that critical region.

The FC was evaluated for all children of the four sites using the AAL, HO, CC, and Power atlases (**Figure 3A**). For each child we identified the possible null rows/columns in the FC matrix due to null time series. When the HO, the CC and the Power atlases were applied, null time courses were obtained in some cerebral regions (**Figure 3B**). Specifically, in the temporal, frontal and parietal lobes, close to brain edges. Subjects with at least one null row/column in the FC matrix were identified in the datasets related to HO, CC, and Power atlases and the critical regions were highlighted for each parcellation scheme. These regions are shown in **Figure 3B**, where they are represented as spheres positioned in the centroid of each atlas region with a radius proportional to the number of subjects (n) presenting that critical region. In order to avoid removing regions that may be potentially interesting for ASD diagnosis, we decided to remove the subjects from HO (n = 3) and CC (n = 3) dataset. Regarding Power atlas, since the number of subjects containing critical regions was too high (n = 130), we decided to remove the regions and not the subjects, leaving 230 regions for the classification analysis. Therefore, multisite analysis was conducted on 190 subjects with AAL and Power atlases and on 187 subjects with HO and CC.

The connectivity analysis was carried out on cortical and subcortical regions, excluding the cerebellum. Since Harvard-Oxford atlas does not already have cerebellum areas, we removed them in the other atlases we used. Cerebellar areas were identified through the corresponding labels for the AAL and through the label generated from the overlap between AAL and CC for CC templates; in Power atlas, the cerebellum was identified from the corresponding MNI coordinates. After the previous selections the number of regions were reduced to 90 for AAL, 110 for HO, 184 for CC and 230 for Power atlases, respectively.

### Correlations Between Altered FC Values and Clinical and Cognitive Measures

We tested the possible correlations of the four functional connections showing the most significant group differences with autism symptom severity and the overall level of functioning, and we found the following significant results in terms of Spearman ρ: negative correlations of the FC between the R hippocampus and temporal fusiform cortex with The ADOS total (ρ = −0.21, p = 0.04) and ADOS severity scores (ρ = −0.24, p = 0.02); a positive correlation of the FC between L inferior frontal gyrus and R frontal operculum cortex with the FIQ (ρ = 0.196, p = 0.049).

### Classification

The L-SVM classification was carried out on the FC measures of the whole dataset using four different atlases. The classification performances are compared in terms of the mean AUC obtained in the leave-one-site-out cross-validation scheme.

The best performance was obtained using the HO atlas, as shown in **Table 4**. The classification results obtained according to the leave-one-site-out cross-validation scheme of data derived with the HO atlas are reported in more detail in **Table 5**, where, in addition to the AUC, also the sensitivity, specificity and accuracy values are shown. In particular, the highest performance was obtained using FC patterns from KKI, NYU, and UCLA samples in the training phase (n = 139) and leaving out the patterns of the UM sample for the validation (n = 48), as shown in **Table 5**.

**Table 4:** ASD vs. TD classification performance: the impact of using different parcellation schemes in the leave-one-site-out cross-validation scheme is shown in terms of mean and standard deviation of AUC. For each atlas, the number of descriptive features (m) is reported.

	Atlas, mean ± std			
Classification	**AAL**	**HO**	**CC**	**POWER**
	(m = 4005)	(m = 5995)	(m = 16836)	(m = 26335)
AUC (%)	72 ± 3	75 ± 5	70 ± 10	64 ± 6

AUC, area under the ROC curve; std, standard deviation; AAL, Automated Anatomical Labeling; HO, Harvard-Oxford; CC, Craddock.

**Table 5:** ASD vs. TD classification performance obtained for the Harvard-Oxford atlas. The classification performances are reported in terms of sensitivity, specificity, accuracy and AUC for each site left out as validation set in the cross-validation scheme. The mean and standard deviation of all figures of merit over the four sites are also reported (the mean AUC and its standard deviation are also shown in **Table 4**).

	L-SVM				
	Leave one site out				
Classification	KKI	NYU	UCLA	UM	mean ± std
Sensitivity (%)	67	48	83	79	69 ± 16
Specificity (%)	75	83	61	75	74 ± 9
Accuracy (%)	71	63	73	77	71 ± 6
AUC (%)	71	75	72	83	75 ± 5

AUC, area under the ROC curve; std, standard deviation.

### Significant Connections

**Figure 4:**
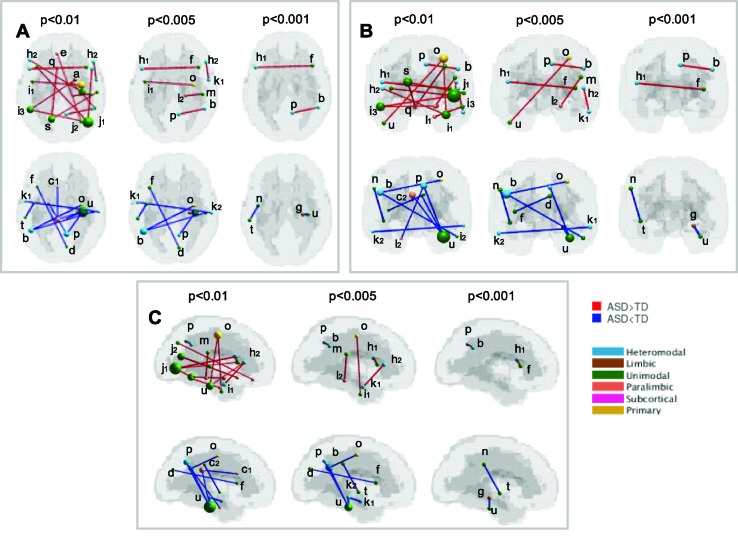
Significant functional connections in the discrimination between subjects with ASD and typical controls obtained using the HO atlas. Altered connections are shown in axial **(A)**, coronal **(B)** and sagittal views **(C)**. In each view, the over-connectivity (top row) and under-connectivity (bottom row) patterns in ASD children in and between the functional Mesulam divisions are shown for different thresholds on significance levels (p < 0.01, p < 0.005, and p < 0.001). The membership of each region to one of the Mesulam division is highlighted by color code applied to a sphere positioned in the region centroid, whose radius is proportional to the number of altered connections involving that region. Lowercase letters are reported to indicate each region, in reference to the reference to the results reported in **Tables 6** and **7**.

The functional connections that contribute the most to the discrimination between subjects with ASD and controls were obtained through a permutation test applied to the dataset of children from all sites together (KKI, NYU, UCLA, and UM), including 187 subjects. The list of relevant functional connections between brain regions are reported in **Tables 6** and **7**, and they are shown in **Figure 4** where over-FC and under-FC patterns are highlighted for different thresholds on p values. The altered functional connections are represented in axial (**Figure 4A**), coronal (**Figure 4B**) and sagittal (**Figure 4C**) views. In the top row of each panel the functional connections which are significantly stronger in ASD relative to TD are depicted, whereas in the bottom row the functional connections which are significantly weaker in ASD relative to TD are shown. Each region is represented as sphere positioned in the region centroid, with a radius proportional to the number of connections involving that region and coloured according to the membership in the six functional Mesulam divisions: heteromodal, unimodal, limbic, paralimbic, subcortical, and primary. This representation facilitates the considerations regarding altered connections in and between functional brain areas.

**Table 6:** List of significantly stronger (ASD > TD) functional connections in ASD children from KKI, NYU, UCLA, UM, obtained for p < 0.01, p < 0.005, and p < 0.001. Beside the Harvard-Oxford labels of the regions defining the connections, lowercase letters are reported in reference to the visual representation of each connection shown in **Figure 4**.

Significant connections					
Harvard-Oxford regions			Mesulam subsystems		p-value
ASD > TD					
R Angular Gyrus (b)	*–*	R Precuneus Cortex (p)	Heteromodal	Heteromodal	<0.001
L Inferior Frontal Gyrus (pars opercularis) (h1)	*–*	R Frontal Operculum Cortex (f)	Heteromodal	Unimodal	<0.001
R Inferior Frontal Gyrus (pars triangularis) (h2)	*–*	R Middle Temporal Gyrus (anterior division) (k1)	Heteromodal	Heteromodal	<0.005
R Precentral Gyrus (o)	*–*	L Inferior Temporal Gyrus (anterior division) (i1)	Primary	Unimodal	<0.005
R Parahippocampal Gyrus (posterior division) (l2)	*–*	R Parietal Operculum Cortex (m)	Paralimbic	Unimodal	<0.005
R Amygdala (a)	*–*	L Inferior Temporal Gyrus (temporo-occipital part) (i3)	Limbic	Unimodal	<0.01
R Inferior Frontal Gyrus (pars opercularis) (h1)	*–*	R Lateral Occipital Cortex (inferior division) (j1)	Heteromodal	Unimodal	<0.01
L Inferior Temporal Gyrus (temporo-occipital part) (i3)	*–*	R Lateral Occipital Cortex (inferior division) (j1)	Unimodal	Unimodal	<0.01
R Lateral Occipital Cortex (superior division) (j2)	*–*	L Frontal Medial Cortex (e)	Unimodal	Paralimbic	<0.01
R Inferior Temporal Gyrus (temporo-occipital part) (i3)	*–*	R Parahippocampal Gyrus (anterior division) (l1)	Unimodal	Paralimbic	<0.01
L Inferior Frontal Gyrus (pars triangularis) (h2)	*–*	R Temporal Fusiform Cortex (posterior division) (u)	Heteromodal	Unimodal	<0.01
R Precentral Gyrus (o)	*–*	R Temporal Fusiform Cortex (posterior division) (u)	Primary	Unimodal	<0.01
R Lateral Occipital Cortex (inferior division) (j1)	*–*	L Frontal Operculum Cortex (f)	Unimodal	Unimodal	<0.01
R Superior Temporal Gyrus (posterior division) (r)	*–*	L Supracalcarine Cortex (s)	Unimodal	Unimodal	<0.01
L Subcallosal Cortex (q)	*–*	L Supracalcarine Cortex (s)	Paralimbic	Unimodal	<0.01

R, right hemisphere; L, left hemisphere.

**Table 7:** List of significantly weaker (ASD < TD) functional connections in ASD children from KKI, NYU, UCLA, UM, obtained for p < 0.01, p < 0.005, and p < 0.001. Beside the Harvard-Oxford labels of the regions defining the connections, lowercase letters are reported in reference to the visual representation of each connection shown in **Figure 4**.

Significant connections					
Harvard-Oxford regions			Mesulam subsystems		p-value
ASD < TD					
R Hippocampus (g)	–	R Temporal Fusiform Cortex (posterior division) (u)	Limbic	Unimodal	<0.001
L Supramarginal Gyrus (anterior division) (t)	–	L Planum Polare (n)	Unimodal	Unimodal	<0.001
L Middle Temporal Gyrus (anterior division) (k1)	–	R Middle Temporal Gyrus (posterior division) (k2)	Heteromodal	Heteromodal	<0.005
R Precentral Gyrus (o)	–	L Angular Gyrus (b)	Primary	Heteromodal	<0.005
R Inferior Temporal Gyrus (posterior division) (i2)	–	L Angular Gyrus (b)	Unimodal	Heteromodal	<0.005
R Precuneus Cortex (p)	–	R Temporal Fusiform Cortex (posterior division) (u)	Heteromodal	Unimodal	<0.005
R Cuneal Cortex (d)	–	L Frontal Operculum Cortex (f)	Unimodal	Unimodal	<0.005
L Cingulate Gyrus (anterior division) (c1)	–	L Cingulate Gyrus (posterior division) (c2)	Paralimbic	Paralimbic	<0.01
R Precuneus Cortex (p)	–	L Parahippocampal Gyrus (anterior division) (l1)	Heteromodal	Paralimbic	<0.01
R Cingulate Gyrus (posterior division) (c2)	–	R Temporal Fusiform Cortex (posterior division) (u)	Paralimbic	Unimodal	<0.01
L Cingulate Gyrus (posterior division) (c2)	–	R Temporal Fusiform Cortex (posterior division) (u)	Paralimbic	Unimodal	<0.01

R, right hemisphere; L, left hemisphere.

## Discussion

The goal of this study was to highlight through machine-learning based techniques possible alterations in the FC of children with ASD in the age range of 6.5–13 years, available within the ABIDE cohort. Several selection criteria were adopted to focus our investigation on a more homogeneous sample of subjects, and thus to reduce the possible sources of variability. Specifically, age, sex, and eye status of the participants are known factors that may introduce heterogeneity in FC. Consequently, this study was focused on male children, in a limited age range, whose rs-fMRI scans were acquired with open eyes. Furthermore, only the four most populated sites were considered. The FC analysis was carried out using different atlases, and machine-learning classifiers were implemented to select the parcellation scheme with the best discrimination performance. Notably, the choice of atlas has an impact on classification performance for two reasons: as the functional signals of the voxels are averaged within a brain parcel, both the region location and its size affect the signal information content and the noise level. The use of the anatomical HO atlas led to a better classification performance with respect to the use of AAL, CC and Power atlases (see **Table 4**). The use of the anatomical HO atlas led to a better classification performance with respect to the use of AAL, CC and Power atlases (see **Table 4**). This result can be explained in terms of a trade-off between the conflicting needs of averaging the functional signals over a non-too-large brain parcels, while keeping acceptable the number of features to classify. A parcellation scheme with a limited number of parcels would generate a manageable number of features to classify, thus avoiding the classifier overfitting problem. By contrast, averaging the functional signal over brain regions that are too large can cause the weakening or disappearance of the signal itself. This trend can be appreciated in the classification performances shown in **Table 4**. In particular, the FC values derived according to the HO atlas have higher discriminating power with respect to those of the AAL atlas, which is characterized by 30% fewer regions, and thus the signals are averaged over larger brain areas and have lower specificity in describing brain functioning. When the CC and Power atlases are implemented, the expected increase in the performance with the increase in the number of parcels does not hold anymore. Despite CC and Power are defined according to functional parcellation schemes and thus potentially more informative than the structural-atlas based ones, the excessive increase in the number of features makes the classifier overfit data and lose its generalization capability, causing the decrease in classification performance instead of the expected increase. For these reasons, the best compromise, at least for the data sample considered in this study, was the implementation of the HO atlas. The binary classification results between subjects with ASD and controls in a leave-one-site-out cross-validation scheme achieved an average accuracy of 0.71 ± 0.06 and an average AUC of 0.75 ± 0.05. The best performance was obtained when the training was carried out on FC patterns of children from KKI, NYU, and UCLA, and the classifier performance was evaluated on data from the UM site, reaching an AUC of 0.83. Within the cross-validation scheme, large variability in the results obtained on the different left-out samples were detected (e.g. AUC in the 0.71–0.83 range), as a result of the differences in demographic, clinical and possible data acquisition variability across sites. This variability suggests caution in interpreting the results, and requires their dedicated replication on larger homogeneous cohorts of subjects. The difference across the sites is also evident in the statistical analysis conducted on the average FC matrices between one site and the others combined together (**Figure S1**). Lot of functional connections in UM control children differ significantly from KKI, NYU and UCLA combined together, probably for different site parameters, linked to scanning protocol used. Specifically, as shown in **Table 2**, the scan duration of time series in UM is longer than the KKI, NYU and UCLA same parameter. Nielsen et al. (24) demonstrated that the scan duration is linked to the classification accuracy, since the longer the time series, the better the performance achieved. The best performance we obtained on the UM site data in the cross-validation scheme is also consistent with the following interpretation: the composition of the samples collected at each single site is not equivalent in terms of the information it provides on the ASD condition. Moreover, none of the four samples we considered is large enough to represent the entire population of children with ASD, which is intrinsically extremely heterogeneous in terms of etiopathogenesis (43), neuroanatomical alterations (44), and phenotypic expression (45). In addition, if the population with ASD was sufficiently represented by these data samples, we would have obtained a lower standard deviation in the leave-one-site-out cross-validation results.

Our rs-fMRI analysis identified both over- and under-FC patterns in the ASD group relative to controls. This result could be interpreted from a developmental perspective (46, 47), considering that both children – in which generally over-FC prevails (15, 48) – and preadolescents/early adolescents – in which under-FC is more frequently reported (9, 11) – are present in our sample. However, other recent studies suggest the coexistence of over- and under-FC in the brain of subjects with ASD, independently of their age (49, 50). The absence of the adult population did not allow us to verify this hypothesis in our sample.

Specifically, we detected increased FC within DMN (between the angular gyrus and the precuneus) in ASD individuals compared with controls, in line with some previous investigation (16, 48). Interestingly, a study that investigated age-related changes in FC by dycothomizing their sample into younger (6- to 9-year olds) and older subjects (10- to 17-year olds) identified reduced FC between DMN nodes in the older group only, and that this FC in the DMN increased with age in the TD controls, but not in the ASD children, providing support for the “developmental disconnection model” of ASD (51).

A stronger connection between anterior and posterior areas of the brain (e.g. middle temporal and inferior frontal; lateral occipital, and frontal operculum) was detected in our sample. Strikingly, the opposite pattern – under-connection between anterior and posterior areas of the brain for ASD subjects – was identified in a recent investigation that has applied deep learning algorithms to the ABIDE dataset (52). Unlike the current study, Heinsfeld and colleagues (52) did not restrict the analysis to a limited age range: therefore, the opposite direction of correlation between antero-posterior regions could be partly ascribable to the different ages of the samples (adults vs. children).

Among the brain regions in which an increase in FC was detected, it is important to consider the inter-hemispheric connections between the inferior frontal gyri – IFG – (i.e. L pars opercularis with R frontal operculum), since these areas are critical for speech expression, that is frequently impaired in ASD individuals, but also for higher-level social cognitive abilities, such as theory of mind and empathy, typically compromised in ASD. Interestingly, even if all the participants of the current study fall into a near-average FSIQ, we detected a significant positive correlation between the level of cognitive functioning as measured by full-scale intelligence quotient and increased left-right IFG FC. Other studies have also found correlations between cognitive abilities and FC in ASD. For example, Reiter et al. (53) found significant under-FC within the DMN and the visual ventral stream in lower-functioning ASD children compared with matched higher-functioning ASD, while Linke et al. (54) showed that reduced interhemispheric connectivity between auditory cortical areas was correlated with lower verbal IQ. Conversely, some investigations did not report any impact of IQ levels on FC results [9, (i.e. Weng 2010 and Salmi 2013)].

An opposite pattern compared to what we have identified, and thus characterized by weak FC in IFG and other language-related brain regions, has been observed in toddlers with ASD, and was correlated with impairment in expressive language ability (56). Under-FC involving interhemispheric Broca’s area was also reported in adolescents with ASD and clear comorbid language impairment (14), suggesting a role of altered FC in communication deficits of subjects with ASD. Of note, in a recent whole-brain meta-analysis of rs-fMRI investigations in ASD, the IFG is one of the few brain regions in which resting-state activity was increased (57).

Although with a lower statistical significance (p < 0.01), increased FC is also displayed within the temporal cortex of subjects with ASD -between the R inferior temporal gyrus and the R parahippocampal gyrus-. Crucially, an increased local FC in these regions was found in high-functioning adolescents with ASD and was correlated with higher core ASD symptom severity (58). Moreover, a similar pattern of local functional over-FC in posterior brain regions including the parahippocampal gyrus was reported in a mixed group of children and adolescents with ASD (59). This regional pattern of over-FC in posterior brain areas involved in visual processing is consistent with preference for local over global visual processing repeatedly observed in individuals with ASD (60, 61).

Importantly, among the under-FC findings, we observed lowered FC between R hippocampus and R fusiform cortex. In line with this finding, the fusiform and the hippocampus – together with the amygdala – belong to the facial memory regions, i.e. structures that are implicated in the memory for faces, an ability particularly impaired in subjects with ASD (62). Alterations in the fusiform–hippocampal cortex emerged also from studies investigating the anatomy (63), the structural connectivity (64), and the FC (65) of individuals with ASD relative to TC. Moreover, insofar as brain–behaviour relationship is concerned, the reduced connectivity between the hippocampus and the fusiform cortex in the ASD group is related to ASD symptom severity (assessed by the Autism Diagnostic Observation Schedule, total and calibrated severity scores, with higher scores indicating greater impairment). Therefore, our results support an impaired connectivity in the brain systems underlying social cognitive skills that is more pronounced in children with more severe ASD core symptoms, suggesting a direct involvement of FC abnormalities in the ASD pathophysiology. Further, the weaker connection between supramarginal gyrus (part of the inferior parietal lobule) and planum polare (part of the superior temporal gyrus) contributed most to differentiating ASD from TD controls. Notably, these regions belong to the DMN, which has been suggested to be involved in social cognition, theory of mind (66, 67), self-evaluation, and introspection, and whose disruption has been consistently reported in subjects with ASD (7, 68, 69). Therefore, reduction in resting state FC in regions of the DMN might underlie some of the core features associated with ASD. Not unexpectedly, other pivotal hub of the DMN, such as the middle temporal gyrus, the parahippocampal gyrus, the posterior cingulate gyrus, the precuneus, and the angular gyrus are part of the weaker connections we found in children with ASD.

Among the weaknesses of the present study is the limited number of subjects in the sample. We focused on children in the age range of 6.5–13 years, thus strongly reducing the number of subjects available in the ABIDE preprocessed sample. In addition, due to the possible additional heterogeneity factors related to gender and eyes status, we restricted the analysis to males whose scans were acquired with open eyes. Only the four more populated sites satisfying all these conditions were considered for the analysis. The choice of applying narrow selection criteria-thus restricting the analysis to a sample of less than 200 subjects- derives from the need of reducing the heterogeneity factors only to those intrinsically related to the ASD condition. In this framework, we could not assess the impact of sex on altered functional connections, due to the exiguous number of female children in the ABIDE cohorts, and to the unbalanced amount of subjects with ASD and controls at each site (see **Figure 1A**). We provided in the Supplementary Materials a confirmation of the fact that the ASD vs. TD discrimination ability of the classifier increases when increasingly stringent selection criteria are applied. The augmented classification performance in the proposed cross-validation scheme corresponds to better generalization capability of the classifier, which is consistent with a reduced heterogeneity in the multisite cohort.

Despite the restriction criteria adopted on the whole sample, the remaining four cohorts show demographic characteristics that are significantly different across sites, as shown in the ANOVA and Kruskal-Wallis analyses carried out on ASD and TD children (**Table 3**). The different characteristics of the cohorts become visible in the ASD vs. TD discrimination results reported separately on each site in the leave-one-site-out cross-validation scheme (see **Table 5**).

In addition to the characteristics of the population analyzed at each site, other effects may have had an impact on the classification results. To demonstrate the strength of the impact of the site provenience on the classification, we reported in the Supplementary Materials the 4-class L-SVM classification performance of the FC patterns of the TD of the four sites, which reaches an accuracy of 0.94. Since site-related heterogeneity factors play an important role in classification results, it might be appropriate to restrict multisite analyses only to sites that present similar characteristics, for example in terms of scan time duration and scanner vendors. Other approaches could be to consider the site as covariate and regress out the multisite variability from the analysis, or to use advanced techniques to filter out site heterogeneity (70). Other multisite trials, related to other brain diseases, recommend a standardization procedure across sites, including, for example, post-acquisition corrections of image artifacts (71).

A possible limitation of this study, which is related to the size of the sample we considered, is the risk of overfitting during the classifier training. The number of FC features derived using a parcellation atlas with N regions scales as ∼N^2^ thus a compromise should be achieved between the desired granularity of the signal localization and the risk of overfitting, which affects the classifier training when the number of features exceeds the number of available cases. As the latter risk affects all the classification experiments in our analyses, regardless the atlas we used, we adopted the linear-kernel SVM classifiers, which have demonstrated robust generalization performances even in case of small training sets with respect to the number of features (39). Feature selection criteria could also be considered to reduce the risk of overfitting; however, better results are not always guaranteed, due to global effects that may influence the FC (23). Whole-brain feature selection approaches based on L-SVM recursive feature elimination (SVM-RFE) may be attempted (72).

Provided these limitations, it is straightforward that the significant altered connections we found are specific of this data sample and therefore not generalizable to female population, to low-functioning individuals, and to subjects with a different age-range. Our analysis suggests the need to collect more populated data samples, which have to be properly stratified in order to reduce the known sources of heterogeneity that may affect the investigation.

## Conclusion

In conclusion, the use of machine learning techniques has allowed the identification of few significant altered functional connections in children with ASD with respect to controls. Despite an average performance of AUC = 0.75 is achieved in ASD vs. control classification in the leave-one-site-out cross-validation scheme, the classification performances obtained on each single site are highly variable, with AUC values in the 0.71–0.83 range. In particular, for one of the samples (UM), subjects with ASD and controls can be very effectively differentiated (AUC = 0.83) by using the FC patterns learned on the other three sites.

In multisite retrospective studies, selecting sites with similar scanning protocol and restricting the FIQ and age ranges of participants is a prerequisite to limit the impact of confounding factors in the results of the analysis. Nevertheless, these restrictions do not guarantee that the populations represented at each site contribute similar information to the analysis, especially in the case of limited numerosity of the sample and highly heterogeneous conditions.

Despite these considerations, the present study highlighted a set of functional connections that are altered in children with ASD with respect to TD controls. Both over- and under-FC patterns have been detected, confirming the coexistence of mixed FC findings not only in ASD subjects in a wide age range (73), but also within a selected, homogeneous sample of ASD children.

## Data Availability

Publicly available datasets were analyzed in this study. This data can be found here: http://fcon_1000.projects.nitrc.org/indi/abide/

## Ethics Statement

The publicly available data resource ABIDE preprocessed (http://fcon_1000.projects.nitrc.org/indi/abide/) has been used in this analysis.

## Author Contributions

SC and AR designed the study; GS, PB, EF, LP, and PO carried out data processing and analysis; FM and SC interpreted the results; GS, AR, and SC drafted the manuscript; GS edited the manuscript; all authors revised and approved the content of the manuscript.

## Funding

The ABIDE preprocessed data have been used in this analysis (24–26). This work has been partially funded by the Tuscany Government (Bando FAS Salute by Sviluppo Toscana, ARIANNA Project), by the National Institute of Nuclear Physics (nextMR project), and by a grant from the IRCCS Fondazione Stella Maris (Ricerca Corrente, and the “5 × 1000” voluntary contributions, Italian Ministry of Health).

## Conflict of Interest Statement

The authors declare that the research was conducted in the absence of any commercial or financial relationships that could be construed as a potential conflict of interest.

## Footnotes

1 http://fcon_1000.projects.nitrc.org/indi/abide/

## References

[1] American Psychiatric Association Diagnostic and Statistical Manual of Mental Disorders. 5th Edn. Arlington, VA: American Psychiatric Association (2013).

[2] Baio J Wiggins L Christensen DL Maenner MJ Daniels J Warren Z Prevalence of autism spectrum disorder among children aged 8 years — autism and developmental disabilities monitoring network, 11 sites, United States, 2014. MMWR Surveill Summ (2018) 67:1–23. 10.15585/mmwr.ss6706a1 PMC5919599 29701730

[3] Sandin S Lichtenstein P Kuja-Halkola R Larsson H Hultman CM Reichenberg A The familial risk of autism. JAMA (2014) 311:1770–7. 10.1001/jama.2014.4144 PMC4381277 24794370

[4] Andrews DS Marquand A Ecker C McAlonan G Using pattern classification to identify brain imaging markers in autism spectrum disorder. In: Pratt J Hall J, editors. Biomarkers in Psychiatry. Current Topics in Behavioral Neurosciences. Springer, Cham (2018). 40. 10.1007/7854_2018_47 29626339

[5] Biswal B Yetkin FZ Haughton VM Hyde JS Functional connectivity in the motor cortex of resting human brain using echo-planar MRI. Magn Reson Med (1995) 34:537–41. 10.1002/mrm.1910340409 8524021

[6] Yerys BE Wallace GL Harrison B Celano MJ Giedd JN Kenworthy LE Set-shifting in children with autism spectrum disorders: reversal shifting deficits on the Intradimensional/Extradimensional Shift Test correlate with repetitive behaviors. Autism (2009) 13:523–38. 10.1177/1362361309335716 PMC3018342 19759065

[7] Kennedy DP Courchesne E The intrinsic functional organization of the brain is altered in autism. Neuroimage (2008) 39:1877–85. 10.1016/j.neuroimage.2007.10.052 18083565

[8] Anderson JS Nielsen JA Froehlich AL Dubray MB Druzgal TJ Cariello AN Functional connectivity magnetic resonance imaging classification of autism. Brain (2011) 134:3739–51. 10.1093/brain/awr263 PMC3235557 22006979

[9] Weng S-J Wiggins JL Peltier SJ Carrasco M Risi S Lord C Alterations of resting state functional connectivity in the default network in adolescents with autism spectrum disorders. Brain Res (2010) 1313:202–14. 10.3389/fpsyt.2016.00205 PMC2818723 20004180

[10] Hull JV Dokovna LB Jacokes ZJ Torgerson CM Irimia A Van Horn JD Corrigendum: resting-state functional connectivity in autism spectrum disorders: a review. Front Psychiatry (2017) 7. 10.3389/fpsyt.2016.00205 28101064 PMC5209637

[11] Assaf M Jagannathan K Calhoun VD Miller L Stevens MC Sahl R Abnormal functional connectivity of default mode sub-networks in autism spectrum disorder patients. Neuroimage (2010) 53:247–56. 10.1016/j.neuroimage.2010.05.067 PMC3058935 20621638

[12] Monk CS Peltier SJ Wiggins JL Weng S-J Carrasco M Risi S Abnormalities of intrinsic functional connectivity in autism spectrum disorders. Neuroimage (2009) 47:764–72. 10.1016/j.neuroimage.2009.04.069 PMC2731579 19409498

[13] Ebisch SJH Gallese V Willems RM Mantini D Groen WB Romani GL Altered intrinsic functional connectivity of anterior and posterior insula regions in high-functioning participants with autism spectrum disorder. Hum Brain Mapp (2011) 32:1013–28. 10.1002/hbm.21085 PMC6870194 20645311

[14] Verly M Verhoeven J Zink I Mantini D Peeters R Deprez S Altered functional connectivity of the language network in ASD: role of classical language areas and cerebellum. NeuroImage Clin (2014) 4:374–82. 10.1016/j.nicl.2014.01.008 PMC3930113 24567909

[15] Supekar K Uddin LQ Khouzam A Phillips J Gaillard WD Kenworthy LE Brain hyperconnectivity in children with autism and its links to social deficits. Cell Rep (2013) 5:738–47. 10.1016/j.celrep.2013.10.001 PMC3894787 24210821

[16] Uddin LQ Supekar K Lynch CJ Khouzam A Phillips J Feinstein C Salience network-based classification and prediction of symptom severity in children with autism. JAMA Psychiatry (2013) 70:869–79. 10.1001/jamapsychiatry.2013.104 PMC3951904 23803651

[17] Plitt M Barnes KA Martin A Functional connectivity classification of autism identifies highly predictive brain features but falls short of biomarker standards. NeuroImage Clin (2015) 7:359–66. 10.1016/j.nicl.2014.12.013 PMC4309950 25685703

[18] Retico A Giuliano A Tancredi R Cosenza A Apicella F Narzisi A The effect of gender on the neuroanatomy of children with autism spectrum disorders: a support vector machine case-control study. Mol Autism (2016) 7:5. 10.1186/s13229-015-0067-3 26788282 PMC4717545

[19] Bosco P Giuliano A Delafield-Butt J Muratori F Calderoni S Retico A Brainstem enlargement in preschool children with autism: results from an intermethod agreement study of segmentation algorithms. Hum Brain Mapp (2019) 40:7–19. 10.1002/hbm.24351 30184295 PMC8022273

[20] Ecker C The neuroanatomy of autism spectrum disorder: an overview of structural neuroimaging findings and their translatability to the clinical setting. Autism (2017) 21:18–28. 10.1177/1362361315627136 26975670

[21] Alaerts K Swinnen SP Wenderoth N Sex differences in autism: a resting-state fMRI investigation of functional brain connectivity in males and females. Soc Cogn Affect Neurosci (2016) 11:1002–16. 10.1093/scan/nsw027 PMC4884321 26989195

[22] Nair S Jao Keehn RJ Berkebile MM Maximo JO Witkowska N Local resting state functional connectivity in autism: site and cohort variability and the effect of eye status. Brain Imaging Behav (2018) 12:168–79. 10.1007/s11682-017-9678-y PMC5628079 28197860

[23] Abraham A Milham MP Di Martino A Craddock RC Samaras D Deriving reproducible biomarkers from multi-site resting-state data: an Autism-based example. Neuroimage (2017) 147:736–45. 10.1016/j.neuroimage.2016.10.045 27865923

[24] Nielsen JA Zielinski BA Fletcher PT Alexander AL Lange N Bigler ED Multisite functional connectivity MRI classification of autism: ABIDE results. Front Hum Neurosci (2013) 7:599. 10.3389/fnhum.2013.00599 24093016 PMC3782703

[25] Di Martino A Yan C-G Li Q Denio E Castellanos FX The autism brain imaging data exchange: towards a large-scale evaluation of the intrinsic brain architecture in autism. Mol Psychiatry (2014) 19:659–67. 10.1038/mp.2013.78 PMC4162310 23774715

[26] Loomes R Hull L Mandy WPL What is the male-to-female ratio in autism spectrum disorder? a systematic review and meta-analysis. J Am Acad Child Adolesc Psychiatry (2017) 56:466–74. 10.1016/j.jaac.2017.03.013 28545751

[27] Gotham K Pickles A Lord C Standardizing ADOS scores for a measure of severity in autism spectrum disorders. J Autism Dev Disord (2009) 39:693–705. 10.1007/s10803-008-0674-3 19082876 PMC2922918

[28] ABIDE Homepage Available at: http://fcon_1000.projects.nitrc.org/indi/abide/.

[29] ABIDE Preprocessed Homepage Available at: http://preprocessed-connectomes-project.org/abide/.

[30] Craddock C Benhajali Y Chu C Chouinard F Evans A Jakab A The Neuro Bureau Preprocessing Initiative: open sharing of preprocessed neuroimaging data and derivatives. (2013). 10.3389/conf.fninf.2013.09.00041

[31] Craddock C Sikka S Cheung B Khanuja R Ghosh SS Yan C Towards automated analysis of connectomes: the configurable pipeline for the analysis of connectomes (C-PAC). Front Neuroinform Conf Abstr Neuroinf (2013) 10.3389/conf.fninf.2013.09.00042

[32] Murphy K Fox MD Towards a consensus regarding global signal regression for resting state functional connectivity MRI. Neuroimage (2017) 154:169–73. 10.1016/j.neuroimage.2016.11.052 PMC5489207 27888059

[33] Craddock RC James GA Holtzheimer PE Hu XP Mayberg HS A whole brain fMRI atlas generated via spatially constrained spectral clustering. Hum Brain Mapp (2012) 33:1914–28. 10.1002/hbm.21333 PMC3838923 21769991

[34] Power JD Cohen AL Nelson SM Wig GS Barnes KA Church JA Functional network organization of the human brain. Neuron (2011) 72:665–78. 10.1016/j.neuron.2011.09.006 PMC3222858 22099467

[35] Chen H Nomi JS Uddin LQ Duan X Chen H Intrinsic functional connectivity variance and state-specific under-connectivity in autism. Hum Brain Mapp (2017) 38:5740–55. 10.1002/hbm.23764 PMC5783325 28792117

[36] Vapnik VN NV The nature of statistical learning theory. Berlin, Heidelberg: Springer-Verlag (1995). Available at: https://dl.acm.org/citation.cfm?id=211359 [Accessed January 15, 2019].

[37] Kassraian-Fard P Matthis C Balsters JH Maathuis MH Wenderoth N Promises, pitfalls, and basic guidelines for applying machine learning classifiers to psychiatric imaging data, with autism as an example. Front Psychiatry (2016) 7:177. 10.3389/fpsyt.2016.00177 27990125 PMC5133050

[38] Mourão-Miranda J Bokde ALW Born C Hampel H Stetter M Classifying brain states and determining the discriminating activation patterns: support vector machine on functional MRI data. Neuroimage (2005) 28:980–95. 10.1016/j.neuroimage.2005.06.070 16275139

[39] Gori I Giuliano A Muratori F Saviozzi I Oliva P Tancredi R Gray matter alterations in young children with autism spectrum disorders: comparing morphometry at the voxel and regional level. J Neuroimaging (2015) 25:866–74. 10.1111/jon.12280 26214066

[40] Metz CE Receiver operating characteristic analysis: a tool for the quantitative evaluation of observer performance and imaging systems. J Am Coll Radiol (2006) 3:413–22. 10.1016/j.jacr.2006.02.021 17412096

[41] Mesulam MM From sensation to cognition. Brain (1998) 121(Pt 6):1013–52. 10.1093/brain/121.6.1013 9648540

[42] Benjamini Y Hochberg Y Controlling the false discovery rate: a practical and powerful approach to multiple testing. (1995) Available at: http://engr.case.edu/ray_soumya/mlrg/controlling_fdr_benjamini95.pdf [Accessed January 15, 2019].

[43] Ronemus M Iossifov I Levy D Wigler M The role of de novo mutations in the genetics of autism spectrum disorders. Nat Rev Genet (2014) 15:133–41. 10.1038/nrg3585 24430941

[44] Chen H Uddin LQ Guo X Wang J Wang R Wang X Parsing brain structural heterogeneity in males with autism spectrum disorder reveals distinct clinical subtypes. Hum Brain Mapp (2019) 40:628–37. 10.1002/hbm.24400 PMC6865602 30251763

[45] Charman T Loth E Tillmann J Crawley D Wooldridge C Goyard D The EU-AIMS Longitudinal European Autism Project (LEAP): clinical characterisation. Mol Autism (2017) 8:27. 10.1186/s13229-017-0145-9 28649313 PMC5481972

[46] Nomi JS Uddin LQ Developmental changes in large-scale network connectivity in autism. NeuroImage Clin (2015) 7:732–41. 10.1016/j.nicl.2015.02.024 PMC4375789 25844325

[47] Uddin LQ Supekar K Menon V Reconceptualizing functional brain connectivity in autism from a developmental perspective. Front Hum Neurosci (2013) 7:458. 10.3389/fnhum.2013.00458 23966925 PMC3735986

[48] Lynch CJ Uddin LQ Supekar K Khouzam A Phillips J Menon V Default mode network in childhood autism: posteromedial cortex heterogeneity and relationship with social deficits. Biol Psychiatry (2013) 74:212–9. 10.1016/j.biopsych.2012.12.013 PMC3710546 23375976

[49] Hahamy A Behrmann M Malach R The idiosyncratic brain: distortion of spontaneous connectivity patterns in autism spectrum disorder. Nat Neurosci (2015) 18:302–9. 10.1038/nn.3919 25599222

[50] Yerys BE Herrington JD Satterthwaite TD Guy L Schultz RT Bassett DS Globally weaker and topologically different: resting-state connectivity in youth with autism. Mol Autism (2017) 8:39. 10.1186/s13229-017-0156-6 28770039 PMC5530457

[51] Washington SD Gordon EM Brar J Warburton S Sawyer AT Wolfe A Dysmaturation of the default mode network in autism. Hum Brain Mapp (2014) 35:1284–96. 10.1002/hbm.22252 PMC3651798 23334984

[52] Heinsfeld AS Franco AR Craddock RC Buchweitz A Meneguzzi F Identification of autism spectrum disorder using deep learning and the ABIDE dataset. NeuroImage Clin (2018) 17:16–23. 10.1016/j.nicl.2017.08.017 29034163 PMC5635344

[53] Reiter MA Mash LE Linke AC Fong CH Fishman I Müller RA Distinct patterns of atypical functional connectivity in lower-functioning autism. Biol Psychiatry Cogn Neurosci Neuroimaging (2019) 4(3):251–9. 10.1016/j.bpsc.2018.08.009 PMC7202917 30343132

[54] Linke AC Jao Keehn RJ Pueschel EB Fishman I Müller RA Children with ASD show links between aberrant sound processing, social symptoms, and atypical auditory interhemispheric and thalamocortical functional connectivity. Dev Cogn Neurosci (2018) 29:117–26. 10.1016/j.dcn.2017.01.007 PMC5664206 28223033

[55] Salmi J Roine U Glerean E Lahnakoski J Nieminen-von Wendt T, The brains of high functioning autistic individuals do not synchronize with those of others. NeuroImage Clin (2013) 3:489–97. 10.1016/j.nicl.2013.10.011 PMC3830058 24273731

[56] Dinstein I Pierce K Eyler L Solso S Malach R Behrmann M Disrupted neural synchronization in toddlers with autism. Neuron (2011) 70:1218–25. 10.1016/j.neuron.2011.04.018 PMC3119852 21689606

[57] Wang W Liu J Shi S Liu T Ma L Ma X Altered resting-state functional activity in patients with autism spectrum disorder: a quantitative meta-analysis. Front Neurol (2018) 9:556. 10.3389/fneur.2018.00556 30087648 PMC6066523

[58] Keown CL Shih P Nair A Peterson N Mulvey ME Müller R-A Local functional overconnectivity in posterior brain regions is associated with symptom severity in autism spectrum disorders. Cell Rep (2013) 5:567–72. 10.1016/j.celrep.2013.10.003 PMC5708538 24210815

[59] Maximo JO Keown CL Nair A Müller R-A Approaches to local connectivity in autism using resting state functional connectivity MRI. Front Hum Neurosci (2013) 7:605. 10.3389/fnhum.2013.00605 24155702 PMC3792552

[60] Happé F Frith U The weak coherence account: detail-focused cognitive style in autism spectrum disorders. J Autism Dev Disord (2006) 36:5–25. 10.1007/s10803-005-0039-0 16450045

[61] Mottron L Dawson M Soulières I Hubert B Burack J Enhanced perceptual functioning in autism: an update, and eight principles of autistic perception. J Autism Dev Disord (2006) 36:27–43. 10.1007/s10803-005-0040-7 16453071

[62] Wilkinson DA Best CA Minshew NJ Strauss MS Memory Awareness for Faces in Individuals with Autism. J Autism Dev Disord (2010) 40:1371–7. 10.1007/s10803-010-0995-x PMC3050057 20300817

[63] Trontel H Duffield T Bigler E Froehlich A Prigge M Nielsen J Fusiform correlates of facial memory in autism. Behav Sci (Basel) (2013) 3:348–71. 10.3390/bs3030348 PMC3992819 24761228

[64] Conturo TE Williams DL Smith CD Gultepe E Akbudak E Minshew NJ Neuronal fiber pathway abnormalities in autism: an initial MRI diffusion tensor tracking study of hippocampo-fusiform and amygdalo-fusiform pathways. J Int Neuropsychol Soc (2008) 14:933–46. 10.1017/S1355617708081381 PMC3298449 18954474

[65] Khan S Gramfort A Shetty NR Kitzbichler MG Ganesan S Moran JM Local and long-range functional connectivity is reduced in concert in autism spectrum disorders. Proc Natl Acad Sci U S A (2013) 110:3107–12. 10.1073/pnas.1214533110 PMC3581984 23319621

[66] Premack D Woodruff G Does the chimpanzee have a theory of mind? Behav Brain Sci (1978) 1:515. 10.1017/S0140525X00076512

[67] Baron-Cohen S Leslie AM Frith U Does the autistic child have a theory of mind? Cognition (1985) 21:37–46. 10.1016/0010-0277(85)90022-8 2934210

[68] Cherkassky VL Kana RK Keller TA Just MA Functional connectivity in a baseline resting-state network in autism. Neuroreport (2006) 17:1687–90. 10.1097/01.wnr.0000239956.45448.4c 17047454

[69] von dem Hagen EAH Stoyanova RS Baron-Cohen S Calder AJ Reduced functional connectivity within and between “social” resting state networks in autism spectrum conditions. Soc Cogn Affect Neurosci (2013) 8:694–701. 10.1093/scan/nss053 22563003 PMC3739917

[70] Pearlson G Multisite collaborations and large databases in psychiatric neuroimaging: advantages, problems, and challenges. Schizophr Bull (2009) 35:1–2. 10.1093/schbul/sbn166 PMC2643967 19023121

[71] Jack CR Bernstein MA Fox NC Thompson P Alexander G Harvey D The Alzheimer’s disease neuroimaging initiative (ADNI): MRI methods. J Magn Reson Imaging (2008) 27:685–91. 10.1002/jmri.21049 PMC2544629 18302232

[72] Retico A Bosco P Cerello P Fiorina E Chincarini A Fantacci ME Predictive models based on support vector machines: whole-brain versus regional analysis of structural MRI in the Alzheimer’s disease. J Neuroimaging (2015) 25:552–63. 10.1111/jon.12163 PMC4388756 25291354

[73] Oldehinkel M Mennes M Marquand A Charman T Tillmann J Ecker C Altered connectivity between cerebellum, visual, and sensory-motor networks in autism spectrum disorder: results from the EU-AIMS Longitudinal European Autism Project. Biol Psychiatry Cogn Neurosci Neuroimaging (2019) 4:260–70. 10.1016/j.bpsc.2018.11.010 30711508

