# Supplementary material for: Evaluation of Altered Functional Connections in Male Children With Autism Spectrum Disorders on Multiple-Site Data Optimized With Machine Learning
Source: Front Psychiatry. 2019 Sep 20;10:620. doi: 10.3389/fpsyt.2019.00620 (PMC6763745; doi:10.3389/fpsyt.2019.00620)
Supplement: Supplementary file 1 [file DataSheet_1.docx]

**Supplementary materials of Spera et. al., *Evaluation of altered functional connections in male children with Autism Spectrum Disorders on multiple-site data optimized with machine learning***

**Estimate of the impact of site and other confounding parameters on ASD vs. control discrimination performance**

1. *Impact of sample selection criteria on ASD vs. control classification*

The ASD vs. control classification performance may be affected by a variety of confounding parameters. We present here the impact on the two-class classification performance of some selection criteria we adopted in this analysis. In particular, the multicenter ABIDE cohort of subjects has undergone increasingly stringent selection criteria, and the ASD vs. control classification results have been reported in Table s1 in terms of mean and standard deviation of AUC and accuracy. The linear-kernel Support Vector Machine (L-SVM) classification with a 10-fold-cross-validation scheme was carried out on the following samples: children (n=396); male children (n=331); male children with eyes open during rest scan (n=263); and on male children with eyes open during rest scan from the larger sites (n=187).

The highest classification performance was obtained adding the site to the selection criteria on sex and eye status, i.e. restricting the classification to the four more populated sites. Specifically, it is evident that restricting the sources of variability there is an increase in the classification performance.

**Table s1.** Classification of ASD and TD subjects obtained for different selection criteria, using a 10-fold-cross-validation scheme.

|  |  | **Sample groups, mean ± std** | | | | |  |
| --- | --- | --- | --- | --- | --- | --- | --- |
| Classification (%) |  | Children,  14 sites | Male children, 14 sites | Male children, open eyes, 10 sites | Male children, open eyes, 4 sites | |  |
| AUC |  | 66 ± 8 | 64 ± 9 | 67 ± 10 | | 73 ± 11 |  |
| Accuracy |  | 62 ± 7 | 62 ± 8 | 63 ± 9 | | 67 ± 10 |  |

***Abbreviations*.** AUC, area under the ROC curve

*b) Site recognizability in the ABIDE multicenter data*

Heterogeneity factors occurring at different sites may have an impact on classification performance. We evaluated the site recognizability in the multicenter ABIDE cohort through the implementation of a multiclass L-SVM classifier and a statistical test on connectivity patterns of TD children from the four most populated sites we have selected for the analysis. The four-class classification was conducted on TD male children with eyes open during the scan from KKI, NYU, UCLA and UM using L-SVM on functional connections using Harvard-Oxford atlas in a 10-fold-cross-validation scheme. A mean classification accuracy of 0.94 demonstrated the strong impact of sites on connections, introducing a variability factor in the classification of ASD and TD subjects.

On the same dataset, a Mann-Whitney test was carried out on the TD children connectivity values between each site and the other sites combined together to identify the significantly different connections. The normality of distributions of connections values for each group was evaluated by Shapiro-Wilk test. The p-values obtained for each connection were corrected with FDR (q≤0.05) (Fig s1): UM is the site with the higher number of significantly different connections from the remaining sites.

The connectivity patterns of subjects from UM are the most different with respect to the ones of the other sites. A possible explanation is in the rs-fMRI scan duration, which is sensibly longer in the UM protocol with respect to the other ones.


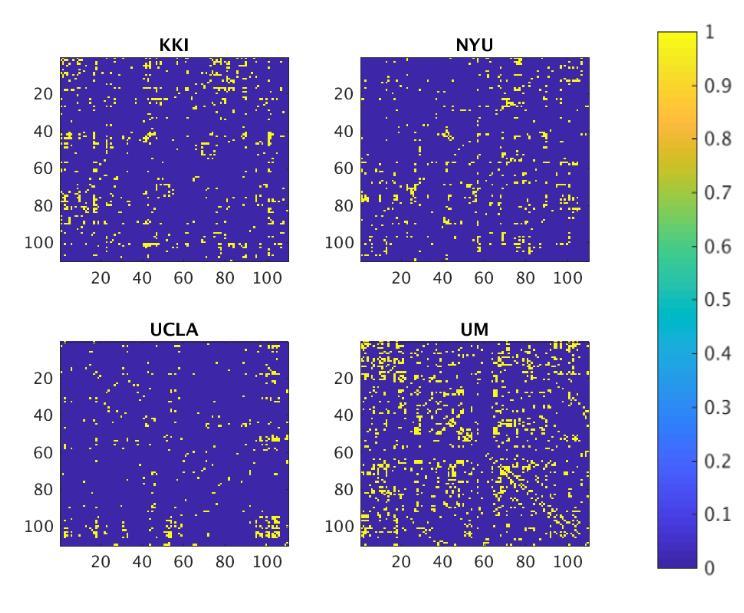


**Fig s1.** Significant functional connections in TD group, distinguishing each site from the remaining sites (FDR, q≤0.05).

**Impact of the global signal regression preprocessing step on the analysis**

The global signal regression (GSR) is a controversial preprocessing technique in rs-fMRI studies. As reported in previous papers, GSR can introduce anticorrelations between rs-state signals -which is visible in Fig.s2-, but it is able to reduce neuronal and non-neural confounds (Murphy *et al*., 2017). Other authors (Chang ang Glover, 2009) demonstrated that anticorrelations can be due also to other noise corrections or have biological origins (Chai *et al*., 2011).

In order to investigate the impact of the use of this preprocessing step in our analysis, a L-SVM classification was performed both on two datasets of functional connectivity values derived for the whole data sample (n=187 subjects), with and without implementing the GSR pre-processing step. The classification of data preprocessed with GSR showed better performance (AUC=0.75 ± 0.05), compared to performance obtained without GSR (AUC=0.71 ± 0.05).


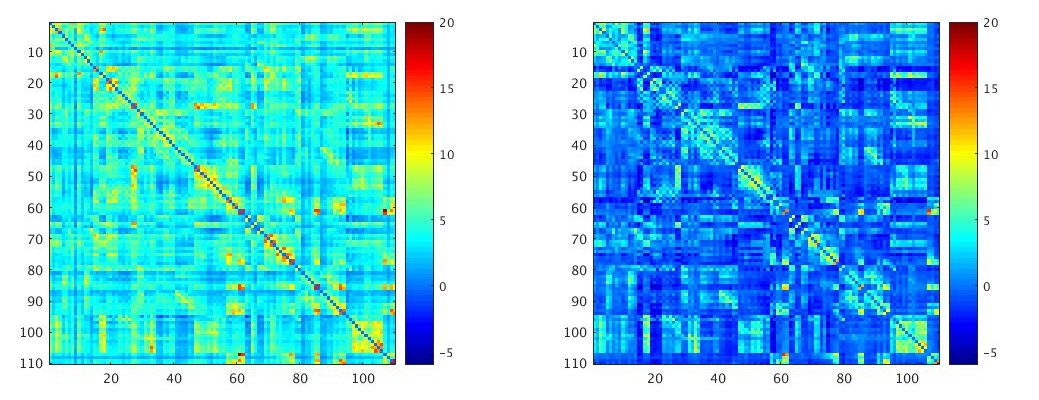


**Fig s2.** Mean functional connectivity in the dataset of 187 subjects, without (left) and with (right) global signal regression preprocessing step. It is evident that anticorrelations between couples of brain areas are introduced.

**References**

Murphy K, Fox MD. Towards a consensus regarding global signal regression for resting state functional connectivity MRI. *Neuroimage* (2017) **154**:169–173. doi:10.1016/j.neuroimage.2016.11.052

Chang C and Glover GH. Effects of model-based physiological noise correction on default mode network anti-correlations and correlations. *Neuroimage* (2009) **47**(4): 1448–1459. doi: 10.1016/j.neuroimage.2009.05.012

Chai XJ, Castañón AN, Öngür D and Whitfield-Gabrieli S. Anticorrelations in resting state networks without global signal regression. *Neuroimage* (2012) **59**(2): 1420–1428. doi: 10.1016/j.neuroimage.2011.08.048
